# Multisite peripheral joint pain: a cross-sectional study of prevalence and impact on general health, quality of life, pain intensity and consultation behaviour

**DOI:** 10.1186/s12891-017-1896-3

**Published:** 2017-12-16

**Authors:** Andrew Finney, Krysia S. Dziedzic, Martyn Lewis, Emma Healey

**Affiliations:** 1 Arthritis Research UK Primary Care Centre, Research Institute for Primary Care and Health Sciences, Keele University, Staffordshire, UK; 2 Keele University, School of Nursing and Midwifery, Clinical Education Centre, University Hospitals of North Midlands NHS Trust, Royal Stoke University Hospital, Staffordshire, ST4 6QG UK

**Keywords:** Cross-sectional population survey, Osteoarthritis, Peripheral joint pain, Multisite joint pain

## Abstract

**Background:**

Research into musculoskeletal conditions often focusses on pain at single sites, such as the knee, yet several studies have previously reported the high prevalence of multiple sites of musculoskeletal pain. The most common form of musculoskeletal condition is arthritis, with osteoarthritis (OA) the most common cause of joint pain in adults 45 years and over. However, there is limited recognition of the prevalence of multisite peripheral joint pain in those either living with or at risk of OA, therefore this study set out to estimate the prevalence of multisite peripheral joint pain in adults 45 years and older, and its impact on several dimensions of health.

**Methods:**

A cross-sectional population survey was mailed to adults (*n* = 28,443) aged 45 years and over from eight general practices in the North West Midlands, United Kingdom (UK). Prevalence rates were established for multisite peripheral joint pain (pain in two or more sites; hands, hips, knees, feet). Impact was measured for general health (SF-12 MCS & PCS), QoL (EQ-5D), pain intensity (0-10 numerical ratings scale) and the number of consultations with a range of health care professionals.

**Results:**

Of 15,083 responders (53%), multisite peripheral joint pain was reported by 54%. Peripheral joint pain was present in *n* = 11,928, of which 68% reported pain in multiple sites. Multisite peripheral joint pain was shown to be significantly associated with reduced physical (Mean difference = −5.9 95% CI -6.3,-5.5) and mental (−2.8 95% CI -3.2,-2.4) components of the SF-12, reduced QoL (−0.14 95% CI -0.15, −0.13), increased pain (+0.70 95% CI 0.62, 0.79) and increased odds of consultations with GPs (OR 2.4 95% CI 2.2, 2.6) and practice nurses (OR 2.6 (95% CI 2.1, 3.2) when compared to single site pain.

**Conclusions:**

Multisite peripheral joint pain is prevalent in the population in adults 45 years and over and has a significant negative impact on several dimensions of health. Health care professionals should consider joint pain beyond the index site in order to address holistic management.

## Background

Two out of three people over the age of 50 years report musculoskeletal pain [1] and it is a common reason for consulting in general practice [2]. The most common form of musculoskeletal disease is arthritis, with osteoarthritis (OA) the most common cause of peripheral joint pain in adults 45 years and over [3]. Research into peripheral joint pain often focusses on pain at single sites, such as the knee or the hip, yet several studies have previously reported the high prevalence of multisite peripheral joint pain in the person [4–7]. There is limited recognition of the prevalence of multisite peripheral joint pain in those either living with or at risk of OA.

Clinical guidelines for OA in the UK provide recommendations for the hands, hips, knees and feet and suggest that many people with OA will have it in multiple sites [8]. The purpose of this study was to determine the prevalence and impact of multisite peripheral joint pain (pain in two or more sites from the hands, hips, knees or feet) in a community dwelling population aged 45 years and over.

## Methods

### Design and participants

A cross-sectional population survey formed part of the ‘Managing Osteoarthritis in Consultations’ MOSAICS study [9]. The postal survey was mailed to 28,443 adults aged 45 years and over registered in eight general practices in the North West Midlands, UK. Prior to mailing, general practitioners (GPs) had the opportunity to screen their lists and exclude ineligible participants e.g. having a psychiatric illness, experienced a recent family bereavement etc. Individuals contacting the research team and not wishing to take part in the survey were tagged in the practices as exclusions and were not contacted again for the study. The population survey used a two-stage mailing process, where eligible participants were sent a letter of invitation to take part in the study and given information about the study. The study was approved by the North West Midlands 1 Research Ethics Committee, Cheshire, UK, as part of the MOSAICS study (ISRCTN number: ISRCTN06984617) [9]. The findings are reported in line with the STROBE guidelines [10].

### Data collection

The survey collected demographic information (age, gender, height, weight) and individual deprivation scores were derived from postal codes (Table 1). Survey questions asked if participants had experienced any pain in or around the hands, hips, knees or feet in the past 12 months, and if yes, to give an average pain intensity score (0-10 numerical ratings scale) for each painful peripheral joint site. General health was measured using the SF-12 physical component scale (PCS) and mental component scale (MCS) [11]. Quality of life (QoL) was measured using the EQ-5D (3-level) [12] and consultation behaviour was measured through asking participants to report if they had consulted a GP, practice nurse, physiotherapist, occupational therapist, podiatrist, hospital specialist, acupuncturist, osteopath or community pharmacist for their joint pain within the past 12 months using a previously validated questionnaire [13].

**Table 1:** Age, Deprivation and BMI characteristics stratified by gender

	All *n* = 15,083 (%)	Females *n* = 8198 (%)	Males *n* = 6885 (%)
Age group (years)			
45-54	3586 (23.8)	1991 (24.3)	1595 (23.2)
55-64	4561 (30.2)	2401 (29.3)	2160 (31.4)
65-74	4038 (26.8)	2138 (26.1)	1900 (27.6)
75+	2898 (19.2)	1668 (20.3)	1230 (17.8)
Deprivation scores (IMD)			
Most Deprived	2948 (19.6)	1591 (19.4)	1357 (19.7)
Middle	8854 (58.7)	4841 (59.1)	4013 (58.3)
Least Deprived	3276 (21.7)	1765 (21.5)	1511 (22.0)
Body Mass Index (BMI)			
Under weight	151 (1.0)	118 (1.5)	33 (0.5)
Healthy weight	5260 (36.8)	3215 (41.6)	2045 (31.1)
Overweight	5750 (40.2)	2697 (34.9)	3053 (46.4)
Obese	3143 (22.0)	1696 (22.0)	1447 (22.0)

BMI classifications devised from NICE (2006) Obesity guidance on the prevention, identification, assessment and management of overweight and obesity in adults and children. NICE clinical guideline 43

### Statistical analysis

Descriptive statistics were used to describe key demographic data and patient reported outcomes. Data were analysed for the 16 possible presentations of peripheral joint pain (including no pain sites as a reference group), before aggregating these into 5 groups (pain in no sites, pain in any one site, any two sites, any three sites and pain in all four sites) for statistical testing. Statistical tests performed were two-tailed, and *p*-values of less than 0.05 were considered statistically significant. The analysis used 95% confidence interval estimates to “infer” beyond the study population. Non-response bias was assessed by aligning patterns of joint pain prevalence and survey returns across age and gender subcategories.

We examined the association between the number of pain sites with social demographic factors (age, gender, body mass index (BMI) and deprivation) and health status measures including the SF-12, EQ-5D and pain intensity. Pain intensity was measured as a mean score across painful peripheral joint sites. Comparisons of consultation behaviour were addressed through descriptive examination and statistical testing of self-reported consultations between study subgroups. Where odds were analysed for pain intensity those with mild pain intensity were compared to those with moderate to severe pain intensity. Mild mean pain intensity was considered to be a score of 1-5 and moderate to severe pain intensity was a score of 6-10 [14].

Statistical examination of association between number of pain sites, social demographic and health status measures were performed through unadjusted (one way ANOVA) analysis. ANOVA was used to calculate an *F*-statistic to determine whether there was a statistically significant overall difference between the mean scores of more than two groups and to then check for a linear trend (via linear contrasts). Multiple linear regression analysis was carried out to evaluate the association between number of pain sites and health status measures, controlling for age, gender, BMI and deprivation. Further multiple linear regression analysis was carried out to investigate the independent effects on health status measures (SF-12, EQ-5D) of, number of pain sites and levels of pain intensity controlling for age, gender, BMI and deprivation. Statistical tests using parametric approaches were generally assumed to be legitimate based on the large study sample. We used logistic regression to analyse the association between the number of pain sites and consultations with general practitioners and practice nurses. Data were analysed using the Statistical Package for Social Sciences version 20 (SPSS, Chicago, IL).

## Results

**Fig. 1:**
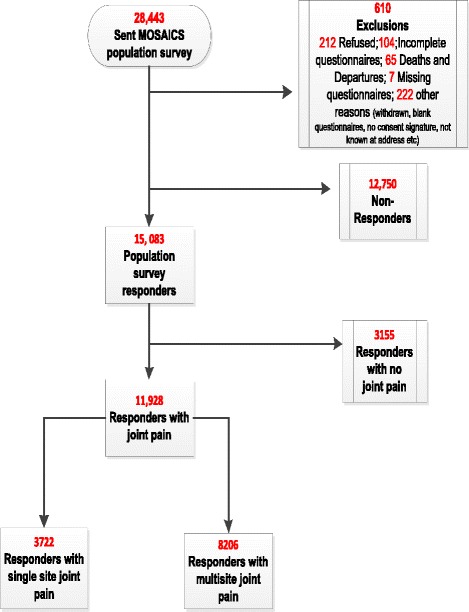
Flowchart of responses to the population survey

Prevalence estimates: Of the 28,443 people who received the population survey *n* = 15,083 (53%) returned the questionnaire, with 11,928 (79%) participants reporting peripheral joint pain (Fig. 1). Table 1 shows the characteristics of survey responders. Overall, 8206 (54%) of responders reported multisite peripheral joint pain. Of those with peripheral joint pain 68% had multisite peripheral joint pain.

### Peripheral joint pain presentations

Peripheral joint pain in the hands, hips, knees or feet was firstly analysed for 16 possible presentations (Table 2). Knee pain was the most prevalent single site. Pain in all four sites was most prevalent. The frequencies of each presentation of peripheral joint pain identified the percentage of participants with a combination of painful joint sites that included the knees 54%, (*n* = 8159), hands 43%, (*n* = 6524), feet 40%, (*n* = 6088) and hips 39%, (*n* = 5957). The combinations of peripheral joint pain in the study population were also descriptively summarised in terms of their mean (SD) age, BMI, SF-12 (PCS & MCS) and EQ-5D scores (Table 2).

**Table 2:** Prevalence of 16 peripheral joint pain presentations and mean age, BMI, the SF-12 PCS and MCS and EQ-5D for all combinations of peripheral joint pain

	Prevalence (%)	Age (Mean & SD)	BMI (Mean & SD)	SF-12 (PCS) (Mean & SD)	SF-12 (MCS) (Mean & SD)	EQ-5D (Mean & SD)
No Pain	20.9	62.6 (11.3)	25.6 (4.0)	51.9 (8.9)	52.5 (8.9)	0.90 (0.15)
Hip pain	4.9	64.0 (11.4)	26.0 (4.2)	47.3 (10.7)	51.2 (9.5)	0.79 (0.21)
Knee pain	9.9	62.5 (11.2)	26.6 (4.3)	48.8 (10.0)	52.5 (8.9)	0.83 (0.17)
Hand pain	5.3	63.7 (10.6)	25.6 (3.9)	50.1 (9.3)	51.3 (9.0)	0.83 (0.19)
Foot pain	4.6	62.4 (11.2)	26.7 (4.11)	49.0 (10.4)	51.2 (9.6)	0.83 (0.19)
Hip & Knee pain	5.6	64.0 (11.0)	27.4 (4.5)	43.3 (11.9)	50.6 (10.5)	0.72 (0.24)
Hip & Hand pain	2.6	65.7 (10.9)	26.3 (4.5)	44.1 (11.8)	50.8 (10.2)	0.74 (0.22)
Hip & Foot pain	2.0	64.4 (10.8)	27.4 (4.7)	44.3 (11.7)	48.7 (10.4)	0.70 (0.25)
Knee & Hand pain	5.7	63.1 (10.9)	26.5 (4.4)	46.0 (11.0)	50.1 (10.1)	0.76 (0.20)
Knee & Foot pain	4.4	62.6 (11.1)	28.1 (4.8)	45.6 (10.8)	50.3 (9.9)	0.74 (0.22)
Hand & Foot pain	3.3	64.2 (11.2)	26.5 (4.2)	45.6 (11.6)	49.4 (10.2)	0.75 (0.20)
Hip, Knee & Hand pain	4.7	65.1 (10.8)	27.1 (4.7)	40.5 (12.1)	49.1 (10.9)	0.65 (0.27)
Hip, Knee & Foot pain	4.4	64.9 (11.6)	28.6 (5.4)	38.6 (12.6)	47.7 (11.3)	0.60 (0.30)
Hip, Hand & Foot pain	2.3	65.4 (11.0)	27.0 (4.8)	42.2 (12.4)	47.6 (10.8)	0.66 (0.27)
Knee, Hand & Foot pain	6.4	64.3 (11.2)	27.8 (5.1)	41.2 (12.1)	48.4 (10.4)	0.66 (0.26)
Hip, Knee, Hand & Foot pain	12.9	66.3 (10.9)	28.4 (5.6)	35.0 (12.2)	45.5 (11.7)	0.51 (0.32)

Using five peripheral joint pain categories trends were investigated and analysed to compare the mean outcomes for age, BMI, deprivation, SF-12 (PCS and MCS) and EQ-5D (Table 3).

**Table 3:** Mean scores for age, BMI, IMD, the SF-12 PCS and MCS and the EQ-5D using the five categories of no joint pain to four sites of joint pain

	No sites of joint pain mean (SD)	Pain in one joint site mean (SD)	Pain in two joint sites mean (SD)	Pain in three joint sites mean (SD)	Pain in four joint sites mean (SD)	Overall difference between groups	Linear trend test
Age	62.6 (11.3)	63.0 (11.1)	63.8 (11.0)	64.8 (11.2)	66.3 (10.9)	*F* = 43.6^a^ *p* < 0.001	*F* = 171.9^b^ *p* < 0.001
BMI	25.6 (4.0)	26.3 (4.1)	27.0 (4.5)	27.7 (5.1)	28.4 (5.6)	*F* = 138.1^a^ *p* < 0.001	*F* = 531.8^b^ *p* < 0.001
IMD	20,685 (8122)	20,985 (8070)	20,324 (8365)	19,657 (8368)	18,561 (8609)	*F* = 33.2^a^ *P* < 0.001	*F* = 114.6^b^ *P* < 0.001
SF-12 (PCS)	51.9 (8.9)	48.8 (10.1)	44.9 (11.5)	40.5 (12.3)	35.0 (12.2)	*F* = 919.2^a^ *p* < 0.001	*F* = 3637.3^b^ *p* < 0.001
SF-12 (MCS)	52.5 (8.9)	51.7 (9.2)	50.1 (10.2)	48.3 (10.8)	45.5 (11.7)	*F* = 190.9^a^ *p* < 0.001	*F* = 752.6^b^ *p* < 0.001
EQ-5D	0.90 (0.15)	0.82 (0.19)	0.74 (0.22)	0.64 (0.27)	0.51 (0.32)	*F* = 1045.5^a^ *p* < 0.001	*F =* 4151.0^b^ *p* < 0.001

^a^= One-way ANOVA used for interval/ratio data with four degrees of freedom. ^b^ = One-way ANOVA with linear contrast with one degree of freedom. IMD sample range 231-32,468 where lower scores indicate the most deprived areas

### Impact on general health and QoL

The independent impact of multisite peripheral joint pain on SF-12 (PCS and MCS) and EQ-5D scores (adjusting for age, gender, BMI and deprivation) was analysed using no sites of joint pain as the reference category. Unstandardized regression coefficients (b) demonstrated reduced and therefore poorer SF-12 PCS scores (−2.68; −5.76; −9.45; −13.89 (*p* = <0.001)), SF-12 MCS scores (−0.85; −2.21; −3.81; −6.45 (*p* = <0.001)) and EQ-5D scores (−0.07; −0.14; −0.23; −0.34 (*p* = <0.001)), for one, two, three and four sites of peripheral joint pain respectively. When all variables within the regression models were standardised to compare individual contributions to the SF-12 (PCS and MCS) and EQ-5D, the variable most significantly associated with reduced general health and QoL was peripheral joint pain in four sites.

### Indicators of impact on pain intensity

Table 4 shows mean pain intensity scores stratified by gender, age, BMI and deprivation. From the summary data it is clear that mean pain intensity increased significantly with each additional peripheral joint pain site. Statistically significant between groups differences in pain scores can be seen across all strata of gender, age, BMI and deprivation (see Table 4).

**Table 4:** Mean pain score from the selected painful sites, stratified by gender, age, BMI and deprivation

	Mean pain (SD) one site	Mean pain (SD) two sites	Mean pain (SD) three sites	Mean pain (SD) four sites	Overall difference between groups	Linear trend test
Gender						
Females	3.44 (2.32)	3.91 (2.11)	4.36 (2.12)	5.29 (2.19)	*F* = 191.9^a^ *p* = <0.001	*F* = 572.2^b^ *p* = <0.001
Males	3.54 (2.30)	3.96 (2.06)	4.45 (2.01)	5.07 (2.15)	*F* = 97.3^a^ *p* = <0.001	*F* = 278.0^b^ *p* = <0.001
Age group *(years)*						
45-54	3.60 (2.25)	3.88 (2.00)	4.28 (2.08)	4.82 (2.17)	*F* = 30.7^a^ *p* = <0.001	*F* = 88.3^b^ *p* = <0.001
55-64	3.38 (2.23)	3.76 (1.98)	4.21 (1.98)	4.94 (2.19)	*F* = 76.5^a^ *p* = <0.001	*F* = 229.1^b^ *p* = <0.001
65-74	3.36 (2.30)	4.01 (2.15)	4.35 (2.02)	5.20 (2.12)	*F* = 93.3^a^ *p* = <0.001	*F* = 275.2^b^ *p* = <0.001
75+	3.71 (2.31)	4.20 (2.24)	4.84 (2.23)	5.82 (2.11)	*F* = 82.7^a^ *p* = <0.001	*F* = 246.3^b^ *p* = <0.001
BMI						
Under weight	3.38 (2.30)	4.06 (2.09)	3.88 (2.11)	5.64 (2.19)	*F* = 4.0^a^ *p* = 0.010	*F* = 10.2^b^ *P* = 0.002
Healthy weight	3.20 (2.26)	3.70 (2.03)	3.91 (2.01)	4.90 (2.18)	*F* = 84.9^a^ *p* = <0.001	*F* = 244.6^b^ *p* = <0.001
Overweight	3.53 (2.26)	3.82 (2.09)	4.39 (2.01)	5.09 (2.15)	*F* = 96.5^a^ *p* = <0.001	*F* = 286.3^b^ *p* = <0.001
Obese	4.03 (2.41)	4.37 (2.05)	4.91 (2.07)	5.54 (2.13)	*F* = 69.0^a^ *p* = <0.001	*F* = 204.3^b^ *p* = <0.001
IMD						
Least Deprived	3.34 (2.16)	3.65 (1.98)	3.98 (1.91)	4.53 (1.83)	*F* = 32.1^a^ *p* = <0.001	*F* = 94.4^b^ *p* = <0.001
Mid Deprived	3.38 (2.29)	3.87 (2.04)	4.30 (2.03)	5.11 (2.18)	*F* = 171.5^a^ *p* = <0.001	*F* = 511.4^b^ *p* = <0.001
Most Deprived	4.08 (2.47)	4.48 (2.26)	5.04 (2.21)	5.91 (2.20)	*F* = 65.5^a^ *p* = <0.001	*F* = 193.8^b^ *p* = <0.001

^a^= One-way ANOVA used for interval/ratio data with four degrees of freedom. ^b^ = One-way ANOVA with linear contrast with one degree of freedom. BMI categories = underweight = <18.5; Healthy weight = 18.5-24.9; Overweight = 25-29.9; Obese = 30+

Using a linear regression model with pain in one site as the reference category, mean pain intensity increased for two, three and four sites (0.34; 0.72; 1.4 (*p* = <0.001) respectively after adjusting for age, gender, BMI and deprivation. When all variables (age, gender, BMI and deprivation) within the model were standardised, the variable most significantly associated with increasing mean pain intensity was peripheral joint pain in four sites.

When analysing the independent effect of number of pain sites and mean pain intensity on the health status measures (SF-12 and EQ5D), mean pain intensity had a greater negative impact than four sites of peripheral joint pain on the SF-12 PCS (−0.38 compared to −0.23), SF-12 MCS (−0.20 compared to −0.14) and the EQ-5D (−0.44 compared to −0.26). Testing for an interaction effect identified that there was a compounding effect of both variables greater than the individual effects for each outcome.

### Consultation behaviour

Of the 11,928 who reported peripheral joint pain, 6549 (54.9%) participants reported attending primary care consultations for their peripheral joint pain over the previous 12 months. Table 5 describes the distribution of consultations stratified by the number of sites of peripheral joint pain. The proportion (%) of people who chose to consult increased for every discipline from one to four sites of joint pain (Table 5).

**Table 5:** The 12-month period prevalence of consultations with Health Care Professionals for peripheral joint pain, stratified by the number of joint sites

Consulted	Pain in one site (*n* = 3722)	Pain in two sites (*n* = 3565)	Pain in three sites (*n* = 2688)	Pain in four sites (*n* = 1953)	Total (*n* = 11,928)
General Practitioner	908 (24.3%)	1291(36.2%)	1263 (46.9%)	1215 (62.2%)	4677 (39.2%)
Practice Nurse	125 (3.3%)	247 (6.9%)	232 (8.6%)	284 (14.5%)	888 (7.4%)
Physiotherapist	489 (13.1%)	609 (17.0%)	568 (21.1%)	466 (23.8%)	2132 (17.8%)
Occupational Therapist	61 (1.6%)	73 (2.0%)	73 (2.7%)	82 (4.1%)	298 (2.4%)
Podiatry/Chiropody	243 (6.5%)	408 (11.4%)	434 (16.1%)	421 (21.5%)	1506 (12.6%)
HospitalSpecialist	428 (11.4%)	534 (14.9%)	554 (20.6%)	492(25.1%)	2008 (16.8%)
Acupuncture	75 (2.0%)	106 (2.9%)	91 (3.3%)	102 (5.2%)	374 (3.1%)
Osteopath/Chiropractor	187 (5.0%)	208 (5.8%)	196 (7.2%)	155(7.9%)	746 (6.2%)
Community Pharmacist	194 (5.2%)	259 (7.2%)	254 (9.4%)	265(13.5%)	972 (8.1%)

Columns show the number and proportion of people who have consulted with each discipline from the number of people who reported peripheral joint pain in one to four sites

Odds of consulting a GP or a practice nurse increased with each additional painful joint site. Using one painful joint site as a reference category the increase in odds of consulting a GP with two sites of peripheral joint pain was 1.66 (1.50, 1.85), three sites 2.52 (2.25, 2.81) and four sites 4.51 (3.98, 5.11). Similarly the odds for consulting a practice nurse increased with two sites of peripheral joint pain to 1.98 (1.58, 2.49), three sites to 2.40 (1.90, 3.03) and four sites 4.30 (3.41, 5.39).

In those consulting a GP the odds increased to 3.68 (3.37, 4.01) in those with moderate to severe pain intensity. In those consulting a practice nurse odds increased to 3.81 (3.22, 4.40) for those consulting with moderate to severe pain intensity. Testing for an interaction effect between the number of peripheral joint pain sites and the level of pain intensity demonstrated no statistically significant interaction effect.

## Discussion

This study used a cross-sectional population survey to determine the prevalence and impact of multisite peripheral joint pain in adults 45 years an older. Multisite peripheral joint pain was shown to be prevalent and associated with poorer general health and QoL. Multisite peripheral joint pain was also shown to have an impact on pain intensity and consultation behaviour. Negative impact was increased with each additional site of joint pain.

The association between pain and general health was maintained when stratifying the population sample by socio-demographic factors (age, gender, BMI, deprivation score) suggesting increased pain intensity and poorer general health are independently associated with peripheral joint pain, and in particular multisite joint pain. Previous research investigating multiple joints, often examined bilateral joint problems [4] or moved beyond joint pain to consider multiple anatomical sites, such as the head or the abdomen etc. [1, 15]. More recent studies have focussed on a primary joint site, with additional sites as comorbidities [16, 17].

Symmons and colleagues previously suggested that multiple sites of joint pain could not be measured by adding together the estimated number of individuals with pain in different joint sites, as this would give an inflated estimate of the overall burden of musculoskeletal pain, instead, studies should consider pain in a number of different sites simultaneously to offer a better estimate and provide useful insights into patterns of pain [18].

In this study a higher proportion of females responded to the survey. The results also demonstrated an increased prevalence of multisite peripheral joint pain in females. This is in keeping with the findings of Peat and colleagues who stressed that multisite lower extremity pain was more common in women [4].

A significant association was found between multisite peripheral joint pain and deprivation scores. A similar finding was reported by Urwin and colleagues who reported that significant differences in deprivation scores were found between those who reported musculoskeletal pain and those that did not [19].

Using a NRS for up to four sites of joint pain to create a mean multisite pain intensity score was novel. There are however more comprehensive measures of pain suitable for cross-sectional surveys than the NRS, such as the Brief Pain Inventory [20]; the McGill Pain Questionnaire [21] or the Chronic Pain Grade [22]. Mallen and colleagues suggest a 3-item prognostic risk tool that addresses pain interference, pain duration and pain in multiple sites should be considered for clinical practice [23]. One potential limitation of this study was the possibility of recall bias with a 12 month time period affecting participants’ ability to accurately self-report information about past pain or consultation behaviour.

We identified an association between peripheral joint pain and self-reported primary care consultations. Comparative studies most often report consultation prevalence from medical records, take their estimates from the general population, and estimate consultation prevalence for OA rather than peripheral joint pain [4, 24, 25]. Consultation data gained via self-report methods, such as this study, may not offer the precision of medical records for conditions with a diagnosis, such as OA, but may provide a better estimate of any consultation in which joint pain is mentioned opportunistically [26, 27].

Consultations for peripheral joint pain with practice nurses were identified as being comparatively low with five times more people with peripheral joint pain consulting their GP than their practice nurse. This suggests that practice nurses are underutilised in the management of musculoskeletal conditions, despite patients with OA wishing to access practice nurses for the management of other chronic conditions [28]. However, one survey in the UK suggested that practice nurses require specific training and education to gain confidence to manage musculoskeletal conditions [29].

The findings of this study suggest people consulting with peripheral joint pain in primary care should be asked about the number of sites of peripheral joint pain they have, irrespective of whether or not they choose only to raise the issue of a primary site. The high prevalence of multisite peripheral joint pain indicates that a significant number of people presenting with single site joint pain will have other painful joint sites. The results suggest that general health and QoL will be dependent on the number of sites of peripheral joint pain. Health Care Professionals should therefore consider joint pain beyond the primary site of presentation in order to address what is predominantly a multisite disease.

## Conclusion

Peripheral joint pain in multiple sites is highly prevalent in those 45 years and over and has a significant negative impact on key dimensions of health. This increasing public health problem suggests that primary care management of peripheral joint pain needs to consider joint pain beyond the primary site of presentation in order to address the management of OA holistically.

## Abbreviations

ANOVA: Analysis of Variance
BMI: Body Mass Index
CI: Confidence Interval
EQ-5D: Euro QoL five dimension
GP: General Practitioner
MCS: Mental Component Score
MOSAICS: Management of Osteoarthritis in Consultations
NRS: Numerical Ratings Scale
OA: Osteoarthritis
OR: Odds ratio
PCS: Physical Component Score
QoL: Quality of Life
SF-12: Short Form 12
STROBE: Strengthening the Reporting of Observational Studies in Epidemiology
UK: United Kingdom
